# An in vitro synthetic biosystem based on acetate for production of phloroglucinol

**DOI:** 10.1186/s12896-017-0376-z

**Published:** 2017-08-08

**Authors:** Rubing Zhang, Wei Liu, Yujin Cao, Xin Xu, Mo Xian, Huizhou Liu

**Affiliations:** 1 CAS Key Laboratory of Bio-Based Materials, Qingdao Institute of Bioenergy and Bioprocess Technology, Chinese Academy of Sciences, Qingdao, 266101 China; 2 University of Chinese Academy of Sciences, Beijing, 100049 China

**Keywords:** In vitro, Acetate, Acetyl-CoA synthetase, Phloroglucinol, High yield

## Abstract

**Background:**

Phloroglucinol is an important chemical, and the biosynthesis processes which can convert glucose to phloroglucinol have been established. However, due to approximate 80% of the glucose being transformed into undesirable by-products and biomass, this biosynthesis process only shows a low yield with the highest value of about 0.20 g/g. The industrial applications are usually hindered by the low current productivity and yield and also by the high costs. Generally, several different aspects limit the development of phloroglucinol biosynthesis. The yield of phloroglucinol is one of the most important parameters for its bioconversion especially from economic and ecological points of view. The in vitro biosynthesis of bio-based chemicals, is a flexible alternative with potentially high-yield to in vivo biosynthetic technology.

**Results:**

By comparing the activity of acetyl-CoA synthetase (ACS) from *Escherichia coli* and *Acetobacter pasteurianus*, the highly active ACS2 was identified in *A. pasteurianus*. Acetyl-CoA carboxylase (ACC) from *Acinetobacter calcoaceticus* and phloroglucinol synthase (PhlD) from *Pseudomonas fluorescens* pf-5 were expressed and purified. Acetate was successfully transformed into phloroglucinol by the combined activity of above-mentioned enzymes and required cofactor. After optimization of the in vitro reaction system, phloroglucinol was then produced with a yield of nearly 0.64 g phloroglucinol/g acetic acid, which was equal to 91.43% of the theoretically possible maximum.

**Conclusions:**

In this work, a novel in vitro synthetic system for a highly efficient production of phloroglucinol from acetate was demonstrated. The system’s performance suggests that in vitro synthesis of phloroglucinol has some advantages and is potential to become a feasible industrial alternative. Based on the results presented herewith, it is believed that in vitro biosystem will provide a feasible option for production of important industrial chemicals from acetate, which could work as a versatile biosynthetic platform.

## Background

Phloroglucinol and its derivatives have been widely applied in pharmaceuticals, cosmetics, textiles, paints and dyeing industries [1]. At present, the chemical synthetic processes are neither cost-effective nor energy saving. Additionally, the production of phloroglucinol and its derivatives using engineered *E. coli* has been investigated by many researchers [2, 3]. However, the productivity and yield are currently still low and hence their industrial production is economically unfeasible. Phloroglucinol is toxic to *E. coli* and its toxicity restrains the further increase of productivity and yield [2]. One of the possible solutions to this problem is to utilize an in vitro biosynthesis technology which does not need the cells and only employs active enzymes. Compared to in vivo living entity-based biosynthetic technology, in vitro synthetic biosystem has broad reaction environment (e.g. high temperature, organic phase catalytic system), free choice of substrate, and potential possibility to solve the problem of the toxicity deriving from the intermediate or final products [4]. The many undesirable side reactions are eliminated in the in vitro biosynthetic processes, thus it becomes possible to achieve about 100% theoretical yield [5]. Moreover, central metabolism which is fundamental to cell survival can be easily re-established through eliminating cellular constraints [6]. Recently, in vitro synthetic biochemistry approaches have been proposed to produce chemicals and proteins [7–9]. In theory, phloroglucinol could be also produced by in vitro synthesis system with the precursor of acetate. Acetate could be converted into acetyl coenzyme A (acetyl-CoA), which can further transform into malonyl coenzyme A (malonyl-CoA) and phloroglucinol.

Acetate had been previously utilized as a feedstock for the production of biochemical or biofuel since it could be transformed into acetyl-CoA. Acetate could be derived from a variety of cheap sources, such as (i) products during syngas fermentation [10], hydrolysis under acid or alkali pretreatment [11] and pyrolysis of lignocellulosic biomass [12], (ii) intermediates from anaerobic digestion of organic wastes [13], (iii) production by methanol carbonylation [14], (iv) methane conversion from natural gas or biogas [15]. It has been demonstrated that *Cryptococcus curvatus* could be able to produce lipid using acetate as a major carbon source [16] and engineered *E. coli* could convert acetate to succinic acid, fatty acids and β-caryophyllene [17–19]. The utilization of acetate as a nontraditional carbon source is becoming one of the most interesting direction in industry biotechnology due to obviously lower cost, sufficient source, no direct competition for food supplies with people, and price stability. However, high concentration of acetate is toxic to cell growth since it damages trans-membrane pH gradients, and which hence destroys internal osmotic pressure, hinders recombinant protein production and inhibits biomass growth [20]. In addition, the formation of the by-products reduces the effective utilization of substrate and also decreases the yield of target product. Considering the advantages of the in vitro biosystem, it is major significant to assimilate acetate via the in vitro synthetic biology.

Acetyl-CoA plays a central role both in catabolism and anabolism, which is a branching point from the substrate carbon into oxidation or biosynthesis pathways [21]. Acetyl-CoA could be also the precursor of many important chemicals, such as fatty acid, succinic acid, 3-hydroxypropionic acid, ethanol, amino acids. Therefore, any biosynthetic process that can utilize acetyl-CoA may become one of the most interesting directions for future biotechnology. It would be important to efficiently convert acetate into acetyl-CoA which is the building block molecule. Until now, there is no report on in vitro biosystem which can make used of acetate as the carbon source to synthetize chemicals.

**Fig. 1:**
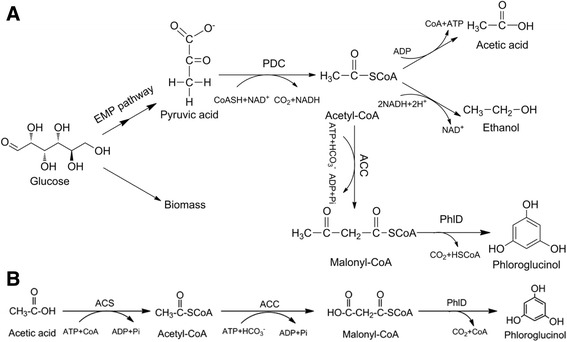
The synthetic pathway of phloroglucinol. A: In vivo process for phloroglucinol fermentation from glucose; B: In vitro process for the transformation of acetic acid to phloroglucinol. The used enzymes are acetyl-CoA synthetase (ACS), acetyl-CoA carboxylase (ACC), phloroglucinol synthase (PhlD), pyruvate dehydrogenase Complex (PDC)

In this work, we attempted to construct an in vitro system for the biosynthesis of phloroglucinol using acetate as the substrate (Fig. 1). With the aim to enhance the utilization ability of acetate, acetyl-CoA synthetases which derived from two different bacteria were screened with high activity. *A. calcoaceticus* was used to clone genes of ACC. The fatty acids of *A. calcoaceticus* is higher than most of the other bacteria, ACC of the bacterium maybe has high activity. *P. fluorescens* Pf-5 which had been analyzed about phloroglucinol synthesis pathway was a source of PhlD. The in vitro synthesis of phloroglucinol was successfully demonstrated. In addition, the process of multi-enzyme catalytic synthesis of phloroglucinol were observed and optimized.

## Methods

### Stain, chemicals and culture conditions

All strains and plasmids used in this study are shown in Table 1. *E. coli* DH5α was used as cloning host and *E. coli* BL21(DE3) (Tiangen, China) was used as expression of recombinant proteins. *A. pasteurianus* DSM 3509 and *A. calcoaceticus* CGMCC 1.6186 were purchased from DSMZ (Brauschweig, Germany) and CGMCC (Beijing, China), respectively. Plasmid pET-28a(+) and Ni-NTA His · Bind Column were purchased from Novagen. PrimeSTAR Max DNA Polymerase (Takara, Japan) was used to amplify genes from plasmid or genomic DNA. Restriction enzymes and T4 DNA ligase (Thermo Scientific, USA) were used for cloning. Commercial enzymes, ATP, coenzyme A and NAD^+^ were from Sigma. Recombinant proteins were concentrated by using the ultrafiltration membrane (Millipore). Bacteria were cultured in Luria-Bertani (LB) liquid medium or LB agar (50 μg/mL of kanamycin was used as the antibiotic for each recombinant *E. coli*).

**Table 1:** Plasmids and strains used in this study

Name	Description
Stains	
*E. coli* K-12	For amplification of gene *acs*
*A. pasteurianus*	For amplification of gene *acs1* and *acs2*
*A. calcoaceticus*	For amplification of four subunits of gene *acc*
Plasmids	
pET-28a(+)	*Kan* ^*r*^ *oripBR322 lacI* ^*q*^ *T7p*
pET28a-acs	pET-28a(+) carrying *acs* from *E. coli* K-12, *Kan* ^*r*^
pET28a-acs1	pET-28a(+) carrying *acs1* from *A. pasteurianus*, *Kan* ^*r*^
pET28a-acs2	pET-28a(+) carrying *acs2* from *A. pasteurianus*, *Kan* ^*r*^
pET28a-accA	pET-28a(+) carrying *accA* from *A. calcoaceticus*, *Kan* ^*r*^
pET28a-accB	pET-28a(+) carrying *accB* from *A. calcoaceticus*, *Kan* ^*r*^
pET28a-accC	pET-28a(+) carrying *accC* from *A. calcoaceticus*, *Kan* ^*r*^
pET28a-accD	pET-28a(+) carrying *accD* from *A. calcoaceticus*, *Kan* ^*r*^
pET28a-phlD	pET-28a(+) carrying *phlD* from *P. protegens*, *Kan* ^*r*^

### Plasmid construction

ACS gene was amplified from the wild-type *E. coli* K-12. *A. pasteurianus* has two putative acetyl-CoA synthetase genes (acs1 and acs2). The genome was extracted from strain *A. pasteurianus* and was used to amplify ACS1 and ACS2 genes. ACS1 and ACS2 were separately cloned into the plasmid pET-28a(+) to produce expression plasmids with a 6xHis tag for purification. The four subunit (accA, accB, accC, accD) genes of acetyl-CoA carboxylase from *A. calcoaceticus* CGMCC 1.6186 were cloned into expression vector pET-28a(+), respectively. The gene encoding sequences of phloroglucinol synthase from *P. fluorescens* Pf-5 were codon-optimized for *E. coli* expression and synthesized by GeneWiz (Suzhou, China). Then phloroglucinol synthase gene was cloned into pET-28a(+) vector with a 6xHis tag for purification, creating plasmid pET28a-phlD. All the forward and reverse primers for the PCR amplification were provided by GeneWiz.

### Enzyme expression and purification

According to the previous protocol [22], all proteins were expressed in *E. coli* BL21(DE3) and then were purified. The single clone grown on LB agar was chosen and inoculated in 20 mL LB seed medium. Seed cultures were used as 1% inoculum in 1 L LB liquid medium; the cells were grown in medium until OD_600_ to 0.65 at 37 °C and then induced by 0.25 mM IPTG. Protein expression was performed under the condition of 20 °C for 12 h. Cells were harvested from 1 L of culture by centrifugation at 4 °C, washed twice using PBS buffer (10 mM Na_2_HPO_4_, 2 mM KH_2_PO_4_, 137 mM NaCl, 2.7 mM KCl, pH 7.4), and re-suspended in 15 ml of 50 mM NaH_2_PO_4_, 300 mM NaCl, pH 7.5. The cells were lysed by sonication on ice and the suspension was centrifuged at 13,000 g for 30 min at 4 °C in order to remove cell debris. Ni-NTA columns were used to purify protein from clear cell lysate following the manufacturer’s protocol. The eluted protein was desalted by ultrafiltration, and stored in the buffer containing 100 mM Tris-HCl, 100 mM NaCl, pH 7.5 at 4 °C. Bradford assay was used to measure protein concentration using bovine serum albumin (BSA) as a standard. The purified proteins were analyzed by SDS-polyacrylamide gel electrophoresis (PAGE) and visualized through Coomassie Blue staining.

### Enzyme activity assays

The specific activity of acetyl-CoA synthetase was measured using Varian Cary50 Bio UV-Visible spectrophotometer at 340 nm according to the previous report [23]. The catalytic activity was determined using the assay mixture at pH 7.4 and 37 °C. The assay mixture (1 mL) contained 1 μmol of NAD^+^, 0.2 μmol of CoA,10 μmol of MgCl_2_, 8 μmol of ATP (pH 7.5), 10 μmol of L-malate (pH 7.4), 100 μmol of Tris-HCl (pH 7.4), 3 units of malate dehydrogenase, 6 units of citrate synthase, 100 μmol of potassium acetate (omitted from controls) and 0.02 nmol of purified protein. The reaction was initiated by adding ATP. The Enzyme activities were calculated according to the speed of NADH accumulation. Kinetic data were further analyzed using software Origin 8.0, and the highest activity of ACS was determined.

The activity of acetyl-CoA carboxylase was determined according to the speed of NADPH oxidation [24]. NADPH concentration was monitored at 365 nm using Varian Cary50 Bio UV-Visible spectrophotometer. The assay mixture (1 mL) contained 0.1 mmol of Tricine-KOH (pH 8.0), 1 μmol of dithiothreitol, 1 μmol of ATP, 2.5 μmol of MgCl_2_, 50 μmol of KCl, 30 μmol of NaHCO_3_, 0.3 μmol of acetyl-CoA (omitted from controls), MCR-C (C-terminal region of malonyl-CoA reductase) [25] and purified acetyl-CoA carboxylase (weight ratio, accA:accB:accC:accD ≈ 1:1.02:1.66:1.08) [26, 27]. Samples were pre-incubated at 30 °C for 1 min to start the reaction, followed by addition of acetyl-CoA, and then incubated at 30 °C after mixing.

The specific activity of the PhlD was measured using the α-ketoglutarate dehydrogenase (KGDH) according to the reported study [22]. CoASH-dependent oxidation of α-ketoglutarate was accompanied by the reduction of NAD^+^ to NADH. The rate of NADH (ε = 6220 M^-1^cm^-1^) formation was monitored at 340 nm. Assay solutions containing 2 μmol α-ketoglutarate, 100 μmol malonyl-CoA (omitted from controls) were added to a premix of 0.2 U KGDH, 0.3 μmol NAD^+^, 30 μg PhlD to a final volume of 1 mL in potassium phosphate buffer (50 μmol, pH = 7.0).

### In vitro synthesis of phloroglucinol

For the enzymatic synthesis of phloroglucinol from acetate, the assay mixture with the final volume of 1 mL contained 10 mM MgCl_2_, 10 mM KCl, 30 mM ATP, 3 mM CoA, 30 mM sodium bicarbonate (NaHCO_3_), 10 mM potassium acetate, 100 mM Tris-HCl pH 7.4, purified ACS2, ACC and phlD, and ampicillin (0.1 mg/mL). To analyze the sensitivity for the concentration of each enzyme, ACS2, ACC, phlD were individually titrated into the reaction system described above. The reactions were executed in 12 mL bottles containing 1 mL of reaction solution. The reaction solution was shaken at 100 rpm in order to exchange substance and energy. The reaction temperature and time were set at 30 °C and 10 h, respectively.

The concentration of acetate and phloroglucinol was analyzed by HPLC. In brief, samples were centrifuged at 12,000 g for 5 min, and the supernatants were filtered using a 0.20 μm nitrocellulose filter and analyzed by a Summit HPLC (Agilent 1200 series, CA, USA) equipped with UV/vis and refractive index detectors. The column (Aminex HPX-87H, 300 mm × 7.8 mm, Bio-Rad, CA, USA) was eluted at 55 °C using 5 mM H_2_SO_4_ as the mobile phase at a flow rate of 0.5 mL/min.

The total phloroglucinol amount was calculated based on the measured concentration by HPLC. The phloroglucinol yield was calculated according to acetate consumption based on the following equivalents: 3 acetate ∼ 3 acetyl-CoA ∼ 3 malonyl-CoA ∼ phloroglucinol.

## Results and discussion

### Kinetic analysis of acetyl-CoA synthetase

**Fig. 2:**
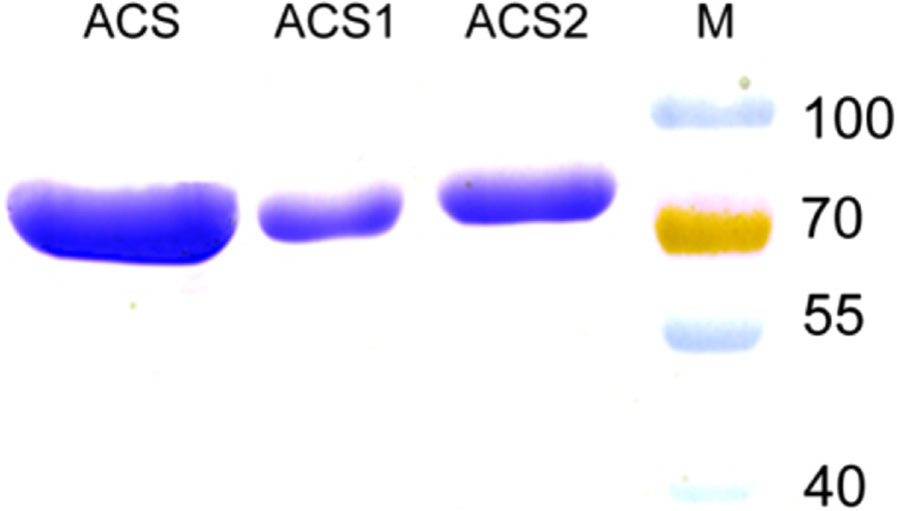
Coomassie brilliant blue-stained SDS-PAGE analysis of purified proteins. Purified ACS, ACS1 and ACS2, Lane M protein molecular weight marker (kD). The positions corresponding to the overexpressed proteins were consistent with their size

**Fig. 3:**
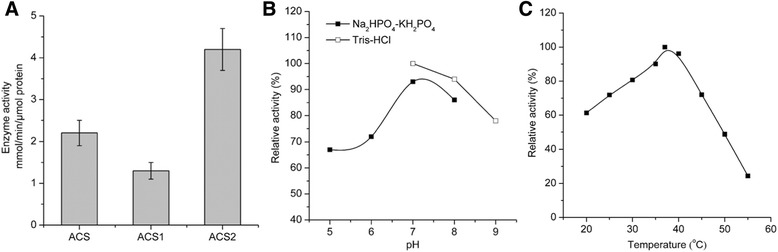
Kinetic analysis of acetyl-CoA synthetase. **a** Enzyme activity comparison of ACS, ACS1, and ACS2. 0.017 nmol (each) of ACS, ACS1, and ACS2 were used to catalyze the reactions at pH 7.4 and 37 °C, respectively. Date represent mean ± standard deviation (*N* = 3). **b** Effects of pH on enzymatic activity of His6-tagged ACS2 proteins. The reactions were performed at 37 °C. 100% corresponds to the enzyme activity at the condition of pH7.0 (Tris-HCl). **c** Effects of temperature on enzymatic activity of His6-tagged ACS2 proteins. The reactions were performed at the condition of pH 7.4 (Tris-HCl). 100% corresponds to the enzyme activity at 37 °C

Three kinds of acetyl-CoA synthetases were analyzed with Coomassie brilliant blue staining after purification (Fig. 2). The kinetic analyses were carried out in vitro by spectrophotometric method. The catalytic activities of ACS, ACS1 and ACS2 were obtained for 2.2, 1.3 and 4.2 mmol/min/μmol protein, respectively (Fig. 3a), showing that ACS2 has the highest affinity for acetate and higher catalyzing efficiency compared with ACS and ACS1 protein. Acetyl-CoA synthetase can assimilate acetate into acetyl-CoA via two irreversible enzymatic steps [28]. Firstly, acetate reacts with adenosine triphosphate (ATP) to produce acetyl-adenosine monophosphate (AMP). Then the reaction of acetyl-AMP with CoA forms acetyl-CoA-releasing AMP. Acetyl-CoA synthetase has high affinity for acetate, which allows it to function at the low concentration of acetate. *Acetobacter aceti* has two putative acetyl-CoA synthetase genes (*acs1* and *acs2*). The *acs1* gene was up-regulated during ethanol-oxidation phases, while the *acs2* gene was significantly up-regulated when cells entered to the acetate-oxidation phase [29, 30]. ACS2 was inferred to play a more important role to assimilate acetate. Many valuable products during biosynthesis pathways can originate from the acetyl-CoA node located in the center of metabolic network, and therefore efficient conversion of acetate to acetyl-CoA is of great significance.

The optimal pH of ACS2 was tested in Na_2_HPO_4_-KH_2_PO_4_ and Tris-HCl buffers with different pH (pH 5, 6, 7 and 8 and pH 7, 8 and 9), respectively. The optimal pH for ACS2 activity was about pH 7.0 (Fig. 3b). To determine the optimal temperature for ACS2, the reactions were incubated at a series of temperatures (20, 25, 30, 35, 37, 40, 45, 50 and 55 °C). The results showed that the highest enzyme activity was identified at about 37 °C and enzyme activity was fairly stable over the temperature range of 25-45 °C (Fig. 3c).

### Effects of individual protein concentration on the phloroglucinol reactions

The ACS2, accA, accB, accC, accD and PhlD were prepared, and the purified proteins were analyzed by SDS-PAGE. Enzyme activity analysis showed that all the purified enzymes were active (Table 2). A sufficient amount of protein was collected in order to carry out in vitro reaction.

**Table 2:** Enzyme used in the in vitro synthesis of phloroglucinol from acetate

Enzyme	EC	Source	Activity (U/mg)	T-Optimun (°C)	pH-Optimun	References
ACS2	6.2.1.1	*A. pasteurianus*	2.91	37	7.0	This study
ACC	6.4.1.2	*A. calcoaceticus*	0.55	30	8.0	[24, 36]
PhlD	2.3.1.253	*P. fluorescens*	1.03	37	7.0	[3, 22]

**Fig. 4:**
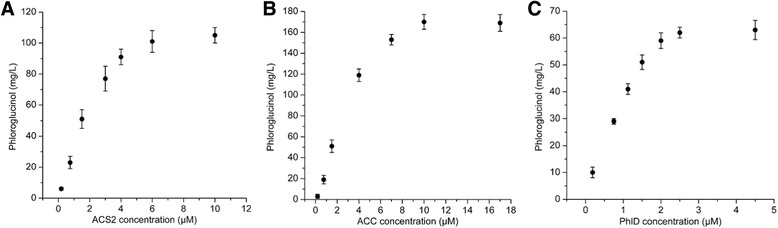
Titration of individual enzyme: **a** ACS2, each assay mixture included 1.5 μM ACC, 1.5 μM PhlD and different concentration of ACS2. **b** ACC, each assay mixture included 1.5 μM ACS2, 1.5 μM PhlD and different concentration of ACC. **c** PhlD, each assay mixture included 1.5 μM ACS2, 1.5 μM ACC and different concentration of PhlD. In all the reactions, substrate and cofactor concentrations were as follows: 10 mM potassium acetate, 30 mM ATP, 3 mM CoA

To analyze the sensitivity of the concentration of each enzyme, ACS2, ACC and PhlD were individually titrated into the in vitro system described above. The concentration of phloroglucinol formation in vitro was quantified when the reaction was terminated at 10 h. Titration results revealed that three enzymes in the biosystem were divided into two categories. ACS2 and ACC enhanced the rate of phloroglucinol synthesis in a dose-dependent manner (Fig. 4a and b). ACC had a more remarkably effect on the in vitro synthesis system, whereas increased ACS2 concentration has relatively less effect. The highest achievable concentration of phloroglucinol was about 170 mg/L when the concentration of ACC varied from 1.5 μM to 10 μM. In contrast, PhlD did not obviously influence the rate of phloroglucinol synthesis, although their individual concentration was varied relative to the reference concentration of 1.5 μM (Fig. 4c). Therefore, the catalyzed reaction of ACC is the rate-limiting step during synthesis of phloroglucinol from acetate. The similar result was also observed during fatty acid synthesis in vivo in the previous study which showed that up-regulation of ACC activity can increase the rate of fatty acid biosynthesis [27]. It is reported that the rate of fatty acid biosynthesis can be increased by 100-fold when ACC was titrated into the cell-free extract from *E. coli* [31], thus high rate of fatty acid synthesis is realizable by increasing the activity or quantity of acetyl-CoA carboxylase. Our results showed the similar importance of acetyl-CoA carboxylase during synthesis of phloroglucinol from acetate in vitro.

### In vitro synthesis of phloroglucinol from acetate

**Fig. 5:**
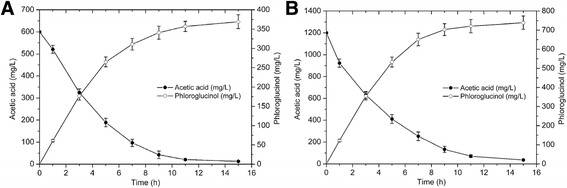
Production of phloroglucinol at a ACS2:ACC:PhlD molar ratio of 3:5:1 (the optimized condition: 6.0 μM ACS2, 10.0 μM ACC and 2.0 μM PhlD). **a** Substrate and cofactor concentrations were 10 mM potassium acetate, 30 mM ATP, 3 mM CoA. **b** Substrate and cofactor concentrations were 20 mM potassium acetate, 60 mM ATP, 6 mM CoA

Based on the results of titration studies, an optimal molar ratio of approximately 3 ACS2 : 5 ACC : 1 phlD (6 μM : 10 μM : 2 μM) was observed under the reaction conditions of 30 °C for 10 h. Finally, 10 mM acetate was converted into 2.93 mM (369.18 mg/L) phloroglucinol at the optimized condition (Fig. 5a). Therefore the optimized molar protein ratio is beneficial to in vitro synthesis system.

The synthesis process required ATP consumption and CoA cycle, and thus the ATP and CoA concentrations might influence the in vitro biosynthetic process. ATP and CoA concentrations would be double in order to extend the duration of phloroglucinol production in the reaction system. As shown in Table 3 and Fig. 5a, the results showed that ATP and CoA concentration have little influence on the phloroglucinol synthesis at the concentration of 10 mM potassium acetate. Under the condition of double concentration, the effect of varying the initial acetate concentration was further examined. Finally, 20 mM acetate was converted into 5.87 mM (739.13 mg/L) phloroglucinol at the optimized condition (Table 2, Fig. 5b), while 40 mM acetate was only converted into 4.17 mM (525.42 mg/L) phloroglucinol. As shown in Table 2, the final amount and yield of phloroglucinol decreased when the initial acetate concentration increased to 40 mM. The results indicated that higher substrate may have many negative impacts on the in vitro synthesis system. The further increase of efficiency and productivity will likely require a coordinated increase at the levels of multiple proteins.

**Table 3:** Effect of initial substrate concentration

Initial acetic acid concentration	Acetic acid measured (mg/L)	Phloroglucinol measured (mg/L)	Weight yield (%)
10 mM (=0.6 g/L)	12.13	376.74	64.09%
20 mM (=1.2 g/L)	36.27	739.13	63.51%
40 mM (=2.4 g/L)	1356.61	525.42	50.36%

In vitro reaction system was incubated with varying amounts of acetic acid. ATP and CoA concentration were 60 mM ATP and 6 mM CoA

In this study, the comparison of the total amounts of transformation and production revealed the high performance of the enzymatic production system. The system shows a much higher yield in comparison to fermentation processes with yields of about 15% [2].

Although the results presented in this work are very promising, several problems should be paid attention. Typically, the productivity was low due to the inhibition of substrate, intermediate or product and the stability of the enzymes was low. The total productivity of the in vitro biosystem is still low (<1 g/L) compared to the reported fermentation processes with productivity of about 4 g/L [2]. The in vitro biosynthetic process for phloroglucinol still needs to further optimize reaction and condition in order to realize the technological applicability. To decrease costs, in vitro biosystem should be operated continuously which can realize the minimal addition of feedstock chemicals. Considering that the consumption of expensive ATP and CoA will increase production costs, low-cost ATP regeneration module need to be designed, and also low-cost and high-stability CoA analogues need to be explored or synthetized [32]. Another possible solution is the construction of efficient enzyme cascade and substrate channeling for in vitro biosynthesis in order to overcome the typical disadvantages for in vitro biotransformation [5, 33] (e.g. the inhibition by the substrate, intermediate, and/or product, and degeneration and diffusive losses of intermediate or cofactors in the reaction). For example, the enzyme cascades were constructed by attaching enzymes to DNA scaffold in the previous work [34], leading to the high reaction rate. It is also important to develop multi-enzyme immobilization cascades and stable enzymes for long-running of in vitro biosynthesis.

## Conclusions

This study was the first successful attempt to synthesize phloroglucinol via in vitro system using acetate as the only carbon source. In comparison to fermentation processes from glucose, a novel approach to produce phloroglucinol from acetate was achieved, which showed a high yield of near 65% through the in vitro biosynthetic process. Considering that the proportion of substrate consumption could be 50% or even more of the total production costs, the overall economy primarily depend on the efficiency of substrate utilization in biochemical processes. In this study, the use of acetate as substrate which is a kind of renewable material provides the potential to reduce the costs of phloroglucinol production. However, some problems need to be solved in the batch production of phloroglucinol. Most notable one is that the used enzymes were not cost effective and recycled. Generally, low costs of enzyme can be realized by discovering stable enzymes suitable for long-term production, designing methods for recycling the enzymes, and developing low-cost methods for protein purification. Therefore, it is suggested that the efforts to develop in vitro biotechnology for the synthesis of bio-based chemicals are further needed.

In this work, the simple artificial multi-enzyme cascade reaction was constructed in an in vitro system based on acetate, and other important bio-based chemicals and bioenergy can also be produced based on the in vitro flexible biosynthesis system. Acetyl-CoA is a central metabolite precursor during biological metabolic processes and can be transformed into other industrial chemicals (e.g. 3-hydroxypropionate and fatty acid) [25, 35], and therefore the in vitro biosynthetic technology based on acetate has the potential to work as a versatile synthetic platform. In brief, in vitro synthesis of phloroglucinol from acetate could be an interesting model system for biosynthesis of other chemicals.

## Abbreviations

ACC: Acetyl-CoA carboxylase
ACS: Acetyl-CoA synthetase
AMP: Acetyl-adenosine monophosphate
ATP: Adenosine triphosphate
IPTG: Isopropyl β-d-thiogalactoside
KGDH: α-ketoglutarate dehydrogenase
PhlD: Phloroglucinol synthase
